# A 14-day repeat dose oral gavage range-finding study of a first-in-class CDI investigational antibiotic, in rats

**DOI:** 10.1038/s41598-018-36690-9

**Published:** 2019-01-17

**Authors:** Katherine Sibley, Jayson Chen, Lee Koetzner, Odete Mendes, Amy Kimzey, Janice Lansita, Ramiz A. Boulos

**Affiliations:** 1 Product Safety Labs, Dayton, NJ United States of America; 2 ToxStrategies, Inc, Katy, TX United States of America; 3 School of Chemical and Physical Sciences, Flinders University, Bedford Park, SA Australia; 4 Antibiotic Development, Boulos & Cooper Pharmaceuticals Pty Ltd, Balcatta, WA Australia

## Abstract

Drug resistant bacteria are winning the fight over antibiotics with some bacteria not responding to any antibiotics, threatening modern medicine as we know it. The development of new, effective and safe antibiotics is critical for addressing this issue. Ramizol, a first-in-class styrylbenzene based antibiotic, is an investigational drug indicated for Clostridium difficile infections (CDI). The objective of this range-finding study was to evaluate the potential general toxicity (based on toxicological endpoints selected) and toxicokinetics of Ramizol in male and female rats that may arise from repeated exposure via oral gavage over a test period of at least 14 days at doses of 50 mg/kg, 500 mg/kg and 1500 mg/kg. There were no mortalities in this study and no Ramizol-related clinical observations. Additionally, there were no changes in mean body weight, body weight gain, food consumption or food efficiency for male and female rats attributable to Ramizol administration. The observed pharmacokinetic behavior showed the presence of Ramizol in plasma at 24 hours post-dosing combined with increasing AUC(0–24) values during the course of this study in groups administered 1500 mg/kg/day, which suggests that at least some dosing groups will show accumulation of compound during repeated dose studies. These toxicology results have shown Ramizol is well-tolerated at very high concentrations in rats and support the further drug development of Ramizol as a first-in-class antibiotic for the treatment of CDI.

## Introduction

The discovery of antibiotics has been one of the greatest discoveries of modern medicine and has played a key role in extending our life expectancy. However, the abuse of their use has contributed to the emergence of multi-drug resistance (MDR) bacteria, predicted to kill over 10 million people by 2050, overtaking cancer as the number one cause of death from disease. At the same time, the scientific bottleneck problem of discovering and developing new antibiotics coupled with the broken economic model for antibiotics has meant several large pharmaceuticals companies have deserted the field. There is now a growing concern that we might end up in the pre-antibiotic era where simple cuts could be deadly and surviving surgery might not be possible, threatening modern medicine as we know it.

The discovery of promising targets remains a major challenge of antibiotic drug discovery. One such target that is the highly conserved in bacteria and not found in the human genome is the mechanosensitive ion channel of large conductance (MscL). MscL channels are transmembrane proteins that has evolved the ability to transduce mechanical stress into an electrochemical response. As such, these channels allow the release of turgor pressure from within, thereby preventing bacteria from lysis. In its fully open state, the channel, which is formed of identical subunits, measures approximately 28 Å, large enough for small proteins, solutes and other drugs to pass through. The in silico design of MscL ligands, has recently led to the discovery of a new class of antibiotics^1^. This came about by developing a spatial map of the exposed oxygen atoms of amino acids, lining the gate of the MscL channel. This three-dimensional map was used for the de novo design of several potential ligands capable of hydrogen bonding to the MscL channel amino acids. The activity of the best hit was then optimized using iterative in silico docking models, which led to the identification of a new class of stilbene-based antibiotics, the activity of which was later confirmed.

Ramizol (registered trademark in Australia) is a first-in-class stilbene-based investigational antibiotic for the treatment of *Clostridium difficile* infections (CDI). It has activity against most drug-resistant Gram-positive bacteria but limited activity against Gram-negative species^1^, reducing the likelihood of collateral damage to the gut microbiota. The reduced activity against Gram-negative bacteria is likely a result of the presence of the lipopolysaccharide-containing outer membrane, which acts as a barrier to drugs. Even though Ramizol is toxic to bacteria, it shows no cytotoxicity in L929 cells (up to 500 µg/mL)^2^ and no haemolytic activity in sheep erythrocytes up to 50 µg/mL, at 25 times the MIC_90_ of *C. difficile*^3^. Moreover, the AMES test established that there is no mutagenic activity due to Ramizol against the test organism *S. typhimurium* for concentrations up to 300 µg/mL^2^. As predicted from in silico modeling, we have previously shown that the antimicrobial potency of Ramizol is dependent on MscL, representing the first success at designing a drug with specificity to MscL (as determined by patch-clamp experiments)^1^. In addition, flow cytometry experiments show that there is no compromise to the integrity of the plasma membrane in bacteria, consistent with the interpretation that Ramizol directly interacts with the channels. Lastly, microscopic imaging using SEM and AFM analysis reveal a significant change in bacterial cell morphology in the presence of Ramizol, with the bacteria flattened and shrunk, which is consistent with MscL channel activation^1^.

Pharmacokinetic comparison of Ramizol (5 mg/kg) delivered intravenously, orally, subcutaneously and intramuscularly in rats has shown the shortest T_max_ of 0.25 ± 0.00 h for IV and the longest T_max_ of 8.00 ± 0.00 h for SC with the T_max_ for oral administration being 1.10 ± 0.24 h^4^. The largest AUC_∞_ was for IV at 692.15 ± 26.15 µgh/mL and the smallest was for oral at 1.38 ± 25.08 µgh/mL while the shortest t_1/2_ was 8.49 ± 0.62 h for IV and the longest was 49.64 ± 8.24 h for SC with a t_1/2_ of 10.50 ± 3.92 h for oral^4^. The absolute bioavailability F was lowest for the oral route at 0.09% while that for SC was the highest at 23.80 ± 4.84% (over 250 x F_oral_). The low oral bioavailability for Ramizol suggest that the drug remained entirely in the gastrointestinal tract akin other non-systemic antibiotics such as vancomycin and fidaxomicin. And while this is ideal for non-systemic infections, other routes of drug administration such as SC would be required for targeting systemic infections to ensure a high enough plasma concentration. At the same time as showing a low likelihood of Ramizol causing adverse effects due to its low bioavailability, we have further eliminated the chance of off-target interaction between Ramizol and mammalian ion channels such as calcium, potassium and sodium ion channels where no significant interaction was observed^5^. Calcium channel L-type has a role in cardiovascular disease, while the N-type mediate calcium influx in cells and directly affect synaptic transmission. The potassium channel [KATP] has a role in modulating blood pressure, while the hERG type and the sodium channel are associated with cardiac arrhythmias. Non-specific interactions with these channels and especially the hERG, are the reason behind the cardiotoxicity of marcrolides, ketolides and fluoroquinolones^6^.

*C. difficile*, which kills over 14,000 people each year in the US alone, is a Gram-positive spore-forming anaerobic bacterium that colonizes the gastrointestinal tract as a result of disturbance to the gut microbiota. Severity of the infection ranges from mild diarrhea to severe life-threating pseudomembranous colitis and toxic megacolon, an inflammation of the colon resulting from the over-growth of *C. difficile*. Consistent with the low bioavailability of Ramizol, the non-systemic nature of the drug is a desirable property for treating *C. difficile* colitis, whereby the drug is present in sufficient concentration to yield a therapeutic effect in the colon. Antibiotic resistance is still rare in *C. difficile* but does occur. In our recent study^3^, Ramizol was fully active against isolates with reduced susceptibility to fidaxomicin, metronidazole and vancomycin, suggesting that pre-existing resistance among *C. difficile* to these agents does not impact Ramizol activity. For Ramizol, the MIC range was ≤0.12–8 μg/mL while for fidaxomicin, vancomycin and metronidazole, the MIC range was wider. Eight isolates were resistant to vancomycin and two to metronidazole, using the EUCAST ECOFF breakpoints (2 μg/ml for both). A breakpoint has not been defined for fidaxomicin. Two of the vancomycin-resistant isolates were noted to be much less susceptible to fidaxomicin (MIC > 8 μg/mL) but were susceptible to Ramizol with MICs of 2 μg/ml and 4 μg/mL. The two strains resistant to metronidazole were susceptible to Ramizol with an MIC of 2 μg/mL and the remaining six strains, which were resistant to vancomycin were susceptible to Ramizol at concentrations from 1 to 8 μg/mL, concentrations similar to the MICs of Ramizol against antibiotic sensitive *C. difficile* strains and MICs against toxin-positive and toxic-negative isolates. Resistance emergence in the presence of Ramizol in *C. difficile* was not observed. The absence of spontaneous mutants in the presence of 4× or 8× the MIC of Ramizol, despite using very high inocula of two *C. difficile* isolates (ATCC 700057 and ATCC 43255) is clinically significant^4^. The data suggest that *C. difficile* has a low chance of developing resistance to the antibiotic in the clinic and the drug is suitable as a monotherapy for the treatment of *C. difficile* infections.

In a *C. difficile* colitis hamster infection model, we have found that Ramizol dosed orally twice a day at 50 mg/kg, saved 43% of the hamsters and when dosed at 100 mg/kg saved 57% of the hamsters, a statistically significant result^4^. In comparison, 100% of hamsters without treatment succumbed to the infection by day 4. By increasing the frequency of doses from twice a day to four times a day, 71% of hamsters treated with a 100 mg/kg dose of Ramizol survived. There was a 2-log decrease in spore count for all treatment groups with vancomycin, Ramizol at 50 mg/kg and Ramizol at 100 mg/kg showing < 1.53 log 10, 1.57 log 10 and 1.83 log 10 spore counts, respectively, compared with to the negative control (vehicle) group showing 3.78 log 10 spore counts per gram stool. It is worth noting that none of the hamsters administered Ramizol suffered any diarrhetic side effects suggesting possibly little or no disruption to the gut flora.

Bacterial infection causes an increase in the reactive oxygen species (ROS) within cells which result in inflammation. We have determined that Ramizol is a potent antioxidant, significantly reducing the generation of intracellular ROS in cells before and after stress^2^. This was observed in L929 cells and in mouse and guinea pig cardiac myocytes with efficacy comparable to α-tocopherol (vitamin E). In cardiac tissue, persistent increases in ROS are associated with pathological remodeling and myocardial dysfunction^7^. Transient exposure to 30 mmol/L H_2_O_2_ results in a significant increase in superoxide production in mouse and guinea pig cardiac myocytes, which was completely attenuated upon the addition 30–50 µg/mL of Ramizol. The antioxidant nature of Ramizol is interesting and could provide a competitive edge for the drug whereby infection-associated inflammation is reduced at the same time as clearing an infection, providing added benefits from treatment.

Current therapeutic agents in clinical development for the treatment of CDI include small molecules such as ridinilazole, biological treatments such as fecal microbiome transplant and SER-109, and immunologic agents such as bezlotomuxab (approved). However, the growing number of deaths from *C. difficile* in the US and elsewhere, and the increasing emergence of resistance in *C. difficile*, warrant the urgent need to develop new, effective and safe therapeutics to tackle CDI.

Here we investigate the 14-day repeat dose range-finding study in rats by oral gavage to determine the potential toxicity from repeated dosing of 50 mg/kg, 500 mg/kg and 1500 mg/kg Ramizol.

## Materials and Methods

The present study was conducted at Product Safety Labs (New Jersey, USA). The animal study protocol was reviewed and approved by the Institution’s Animal Care and Use Committee (IACUC) composed of Jamie Boulet, Jennifer Durando, Carol Soden, Marlene Barton, Barbara Rose, Laura Conour and Matthew Notta. All animal procedures were conducted in compliance with the Guide for the Care and Use of Laboratory Animals^8^, in an Association for Assessment and Accreditation of Laboratory Animal Care International (AAALAC)-accredited, USDA compliant facility.

### Drug preparation

Ramizol, as the ammonium salt, (Boulos & Cooper Pharmaceuticals Pty Ltd) was received and stored at ambient conditions. The powder was dissolved in sodium hydroxide (1.0 M) and then diluted in sodium phosphate buffer (0.1 M, pH 7.4) and adjusted to pH 7.4 with hydrochloric acid (0.5–5 M). The formulations containing 5, 50 and 150 mg/mL concentrations of Ramizol were stirred at ambient temperature until a visually homogeneous mixture was achieved. The mixture was then subjected to filtration with a 0.45 or 0.2 μm filter system. The formulations were prepared weekly, divided into daily aliquots, and stored in a refrigerator for up to 8 days. The aliquots were equilibrated to ambient temperature and vortexed to ensure homogeneity before dosing.

### Dose calculations and dosing

Individual doses were calculated based on the most recent weekly body weights and were adjusted each week to maintain the targeted dose level for all rats (i.e., mg/kg/day). All doses were administered volumetrically at 10 mL/kg. The control group received the vehicle only, at the same dose volume as the test animals.

Each animal was dosed by oral intubation. For all animals, dose administration was daily (7 days/week) for a period of at least 14 days. The dose mixtures were maintained on a magnetic stir plate during dose administration. The first day of administration was considered Day 1 of the study. Dosing was at approximately the same time each day (±2 hours). Residual dose preparations were properly discarded following daily administration and sampling.

### Drug stability and dose preparations

Neat Ramizol and dose preparations were sampled at least once. A sample of Ramizol (neat) was collected at the beginning and end of the in-life phase. Additional samples were collected on Days 7 and 14 and analyzed for verification of dose preparation stability.

Prior to initial dosing, formulations of each concentration were prepared according to the same procedures described previously. Samples from these preparations were collected from the top, middle, and bottom of each concentration of Ramizol that was prepared in the control vehicle. Samples of the vehicle control were collected from the middle of the container only. The dose preparations were sampled at the beginning for verification of concentration at each dose level.

Upon sampling, dose preparations and neat Ramizol were stored in a refrigerator. The refrigerated samples described above were sent to Product Safety Labs Analytical Services for analysis of dose preparations and neat Ramizol samples. All samples were stored and maintained in a refrigerator prior to analysis. An aliquot of Ramizol served as the reference standard. The analytical test method methodology followed that previously published^5^.

### Animals and housing conditions

CRL Sprague-Dawley CD^®^ IGS rats (Charles River Laboratories, Inc.) used for the study were approximately eight weeks old at initiation. After acclimating to the laboratory environment for twelve days, the rats were examined for general health and weighed before randomly assigned, stratified by body weight, to test groups on the day of study start. Forty-eight animals were used for the study (24 main test +24 toxicokinetic satellite) divided in 8 Groups, which consisted of 4 main test groups and 4 toxicokinetic satellite groups, with each group made of 3 males and 3 females (nulliparous and non-pregnant). The animals weighed 233–263 grams (males) and 183–226 grams (females); the weight variation did not exceed ± 20% of the mean weight for each sex.

The animals were individually housed in suspended stainless steel cages which conform to the size recommendations in the latest Guide for the Care and Use of Laboratory Animals^8^.

The animals were conditioned to the housing facilities for twelve days prior to testing. Body weights and clinical observations were recorded at least two times prior to study start. Animal were fed 2016CM Certified Envigo Teklad Global Rodent Diet^®^ (Envigo Teklad, Inc.) ad libitum during acclimation and throughout the study. Filtered tap water was available ad libitum from an automatic watering access system.

### Selection of dose levels

Three male and three female test animals were randomly assigned to each of the following test and satellite groups as in Table 1.

**Table 1:** Administered doses to animals in the different groups.

Group	No. Animals/Group (M/F)	Target Dose Level (mg/kg/day)	Dose Concentration^a^ (mg/mL)
Main Test			
1	3/3	Vehicle Control^b^	0
2	3/3	50	5
3	3/3	500	50
4	3/3	1500	150
Toxicokinetic Satellite			
5	3/3	Vehicle Control^b^	0
6	3/3	50	5
7	3/3	500	50
8	3/3	1500	150

^a^Appropriate concentrations of Ramizol in vehicle to achieve the target dose level. ^b^Sodium phosphate buffer (0.1 M, pH 7.4). Dose volume is 10 mL/kg/day.

The dose levels of 0 (vehicle control), 50, 500, and 1500 mg/kg/day of Ramizol were based on results from a study where hamsters with a lethal *C. difficile* colitis infection were treated with Ramizol at 100 mg/kg BID^5^. A 500 mg/kg/day dose level was therefore used as the intermediate dose, 1/10 of that dose as the low dose and x3 times that dose was used as the high dose. Based upon a preliminary tolerability test, the high dose was a tolerable dose with this formulation and test system and was not expected to cause marked toxicity. The intermediate and low dose levels were selected to derive a dose-response for any effects observed.

### Experimental observations (clinical observations, body weight, and food consumption)

All animals were observed at least twice daily for viability. Cage-side observations of all animals were performed daily during the study. All findings were recorded. For the main test animals, prior to the first treatment with Ramizol on Day 1 and approximately weekly thereafter, a detailed clinical observation was conducted while handling the animal, generally occurring on days that the animals were weighed and food consumption measurements taken. Potential signs noted included, but were not limited to: changes in skin, fur, eyes, and mucous membranes, occurrence of secretions and excretions and autonomic activity (e.g., lacrimation, piloerection, pupil size, unusual respiratory pattern). Likewise, changes in gait, posture, and response to handling, as well as the presence of clonic or tonic movements, stereotypies (e.g., excessive grooming, repetitive circling), or bizarre behavior (e.g., self-mutilation, walking backwards) were also recorded.

Individual body weights were recorded at least two times during acclimation. All test animals were weighed on Day 1 (prior to study start) and approximately weekly thereafter (intervals of 7 days ±1). The main test animals were weighed prior to sacrifice. Body weight gain was calculated for selected intervals and for the study overall.

Individual food consumption was measured and recorded to coincide with body weight measurements for the main test animals. Food efficiency was calculated and reported.

### Toxicokinetic

At the first and last doses, blood samples were collected from the satellite animals (Groups 5–8) for toxicokinetic sample analysis. Blood from Ramizol-treated satellite animals was sampled at six time-points (0.5, 1, 2, 4, 7, and 24 hours) after dosing. Vehicle control satellite animals were sampled once (approximately 3 hours) after dosing on the same days. Approximately 240 μL of blood was collected in capillary blood collection tubes treated with heparin. The blood was then transferred to tubes containing K_2_EDTA and kept on ice until centrifuged. Following centrifugation, the plasma was transferred to clean tubes and stored at approximately −80 °C until transferred. Samples were sent on dry ice to Symbiotic Research and kept frozen before analysis. Toxicokinetic data evaluation was performed by Product Safety Labs. Toxicokinetic satellite animals (Groups 5–8) were not evaluated further.

A high-performance liquid chromatography with ultraviolet detection (HPLC-UV) method for the quantitation of Ramizol was developed and validated. The validated method was used for bioanalytical sample analysis of Ramizol in Sprague Dawley rat plasma, with K_2_EDTA as an anticoagulant. External calibration standards for the quantitation of Ramizol were prepared in 1:1:2 (acetonitrile:methanol:water). Control rat plasma was fortified with Ramizol and sets of quality control (QC) samples were prepared. Ramizol was quantitatively extracted from 50 μL of rat plasma sample with 4% formic acid in acetonitrile. The mixture was vortexed, sonicated, and centrifuged. An aliquot of the supernatant was mixed with the same volume of 1:1:2 (acetonitrile:methanol:water) and injected onto an Agilent HPLC system. Ramizol was separated by a Waters Xselect HSS T3 3.5 μm 2.1 × 100 mm column and the HPLC effluent was monitored by a Diode Array Detector (DAD) at 320 nm. The total run time was 13.0 min. A total of 4 analytical batches were run to analyze all 228 bioanalytical samples.

### Clinical pathology (hematology and serum chemistry)

Clinical pathology was performed on all main test animals (Groups 1–4) for blood chemistry and hematology. The animals were fasted overnight prior to blood collection. Blood samples were collected via sublingual bleeding under isoflurane anesthesia prior to terminal sacrifice. Approximately 500 μL of blood was collected in a pre-calibrated tube containing K_2_EDTA for hematology assessments. The whole blood samples were stored under refrigeration and shipped on cold packs. Approximately 1000 μL of blood was collected into a tube containing no preservative for clinical chemistry assessments. These samples were centrifuged in a refrigerated centrifuge, and the serum was transferred to a labeled tube. Serum samples were stored in a −80 °C freezer and shipped frozen on dry ice. All samples were shipped to DuPont Haskell Global Centers for Health and Environmental Sciences.

### Organ weights, gross necropsy, and histopathology

At terminal sacrifice, all main test animals (Groups 1–4) were euthanized after blood collection for clinical pathology. All animals were subjected to a gross necropsy, which included examination of the external surface of the body, all orifices, and the thoracic, abdominal and cranial cavities and their contents. All gross lesions were recorded. The liver, heart, and kidneys (combined) of the main test animals were weighed.

The following organs and tissues from all animals were preserved in 10% neutral buffered formalin: stomach, duodenum, ileum with Peyer’s patches, colon, cecum, liver, heart, kidneys (combined), lungs and bone marrow (from femur).

Histological examination was performed on the preserved organs and tissues of the main test animals from both the control and high dose groups (Groups 1 and 4, respectively). In addition, gross lesions of potential toxicological significance noted in these test groups at the time of terminal sacrifice were also examined. The fixed tissues were trimmed, processed, embedded in paraffin, sectioned with a microtome, placed on glass microscope slides, stained with hematoxylin and eosin and examined by light microscopy. Slide preparation was performed at Histoserv Inc. Histological assessment was performed at Product Safety Labs by a board-certified veterinary pathologist.

### Data analysis

Product Safety Labs performed statistical analysis of all data collected during the in-life phase of the study as well as organ weight data. DuPont Haskell Global Centers for Health and Environmental Sciences provided analysis of clinical pathology results to Product Safety Labs. The use of the word “significant” or “significantly” indicates a statistically significant difference between the control and the experimental groups. Significance was judged at a probability value of p < 0.05. Male and female rats were evaluated separately.

For all in-life endpoints that are identified as multiple measurements of continuous data over time (e.g. body weight, body weight gain, food consumption, and food efficiency), treatment and control groups were compared using a two-way analysis of variance (ANOVA), testing the effects of both time and treatment, with methods accounting for repeated measures in one independent variable^9^. If a significant interaction effect was observed between treatment and time, further analysis of the p value for each individual factor were conducted ultimately by a post hoc multiple comparisons test (e.g. Dunnett’s test) of the individual treated groups to control^10,11^. If warranted by sufficient group sizes, all endpoints with single measurements of continuous data within groups (e.g. organ weight and relative organ weight) were evaluated for homogeneity of variance^12^ and normality. Where homogenous variances and normal distribution was observed, treated and control groups were compared using a one-way ANOVA. When one-way ANOVA was significant, a comparison of treated groups to control was performed with a multiple comparisons test (e.g. Dunnett’s test). Where variance was considered significantly different, groups were compared using a non-parametric method (e.g. Kruskal-Wallis non-parametric analysis of variance)^13^. When non-parametric analysis of variance was significant, a comparison of treated groups to control was performed (e.g. Dunn’s test)^14^. Statistical analysis was performed on all quantitative data using Provantis^®^ version 9, Tables and Statistics, Instem LSS, Staffordshire UK.

Toxicokinetic parameters were estimated using WinNonlin 7.0 (Certara LP, Princeton, NJ). Parameters included C_max_, T_max_, AUC_(0–24)_, V_d_, Cl, k_en_ and k_el_ and elimination T_1/2_. When appropriate, statistical analysis of toxicokinetic data and parameters was performed using Graphpad Prism.

For the clinical pathology, significance was judged at a probability value of p < 0.05. Males and females were analyzed separately (Provantis TM version 8, Tables and Statistics, Instem LSS, Staffordshire, UK).

## Results

### Drug stability and dose preparation analysis

Ramizol was stable under the conditions of storage during the course of this study. Results of the stability analysis of the neat Ramizol were 104.6% of the target concentration on Day 0 (initial) and 104.1% on Day 6. The difference in the neat Ramizol concentration over the course of the study was of −0.4%, and the overall stability was determined to be 99.6% (Table S1).

Dose preparation samples were collected from week 2 preparations on Days 7 and 14 and compared to assess sample stability under refrigeration. The results for the stability samples were 85.7, 101.3, and 104.3% on Day 7 and 91.4, 99.3 and 101.6% on Day 14 for Groups 2–4, respectively. The difference in the sample concentrations were 6.7, −2.0, and −2.7% for the overall stability of 106.7, 98.0, and 97.3% for Groups 2–4, respectively (Table S2). The dose preparations were considered to be stable under refrigeration over a period of 7-days.

Homogeneity and concentration verification analysis of the Day 0 dose preparations resulted in a relative standard deviation (RSD) of 2.2, 1.7, and 0.7% for Groups 2–4, respectively. Mean values of the top, middle, and bottom samples were 105.5, 111.1, and 101.5% of the target concentrations of 5, 50, and 150 mg/mL in Groups 2–4, respectively (Table S3). Ramizol was homogenously distributed in the dose mixture at all concentrations.

### General clinical observations

There were no mortalities in this study and no Ramizol-related clinical observations (cage-side or detailed clinical observations) for any main test animal during the study.

Incidental cage-side observations in the toxicokinetic satellite animals consisted of slight alopecia of the head in 1/3 Group 7 males and slight to moderate alopecia of the right or left forelimb in 1/3 Group 6 females. These observations were due to excessive grooming habits occasionally observed in rats and not related to Ramizol administration. Moreover, a clear dose response was not observed, and a similar finding was not observed in the main study rats.

### Body weight and food consumption

There were no changes in mean body weight or body weight gain attributable to Ramizol administration. Mean weekly body weights and mean daily body weight gain for male and female rats in Groups 2–4 and 6–8 were comparable to their respective controls throughout the study. There were no changes in mean daily food consumption or food efficiency attributable to Ramizol administration. Mean daily food consumption and mean food efficiency for male and female rats in Groups 2–4 and 6–8 were comparable to their respective controls throughout the study (results not shown).

### Toxicokinetic results

Two samples yielded assay results below the lower limit of quantitation (BLQ), which were replace by zero; no results were above the upper limit of quantitation. This number of BLQ values did not interfere with data analysis. One value met the statistical definition of an outlier (i.e., a criterion score using Dixon’s test) and was excluded. Exclusion of one value did not interfere with data analysis. Quality control results varied between bioanalytical batches, with the first batch below 70% of target. Re-analysis of the first batch was not possible, as prior analysis using a different method had consumed a substantial amount of these early samples. This affected the 0.5, 1 and 2 hour time points in the initial (Days 1, 2) samples for all groups. Accordingly, the time course values for these time points may be underestimated. The impact on calculated parameters is likely modest, with the exception of maximal concentrations during initial samples for Group 6 and entry rates for all groups during initial samples; these parameters could be substantially underestimated. However, increasing the estimates for these two parameters would only exaggerate the effects currently seen in the data. Therefore, no impact is likely.

**Figure 1:**
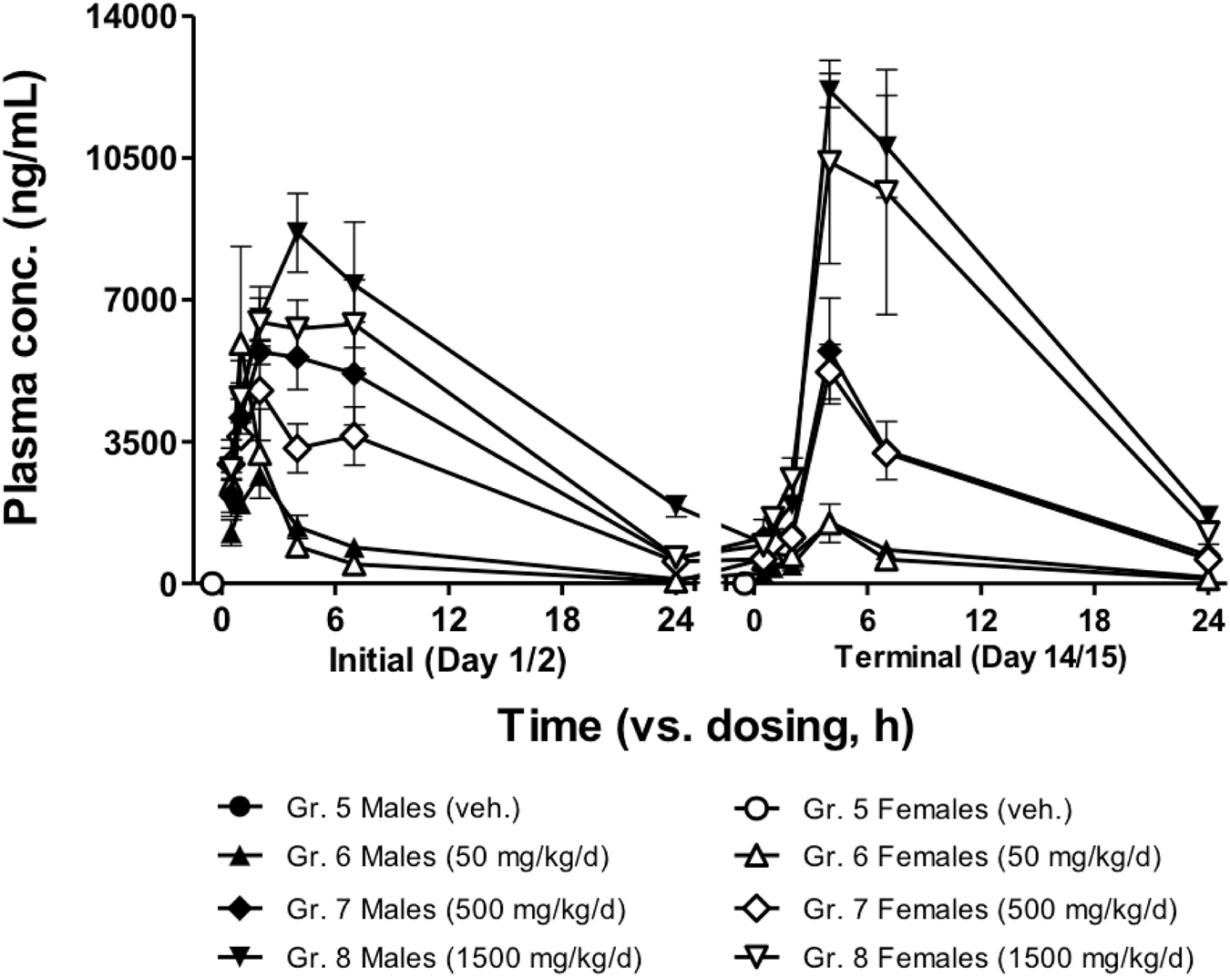
Bioanalytical results from satellite animals.

A time course plot of the data showed differences between dosing groups (Fig. 1), although the increases in plasma concentration did not appear to be proportional to dose. The data also reveal detectable concentrations at 24 hours post-dose; this indicates that accumulation could occur with daily dosing. Comparing initial and terminal (Days 14, 15) samples suggests a treatment-dependent accumulation, with the most obvious increase in Group 8 (1500 mg/kg/day). A possible decrease at peak was apparent in Group 6 (50 mg/kg/day). No obvious male-female differences were observed.

**Figure 2:**
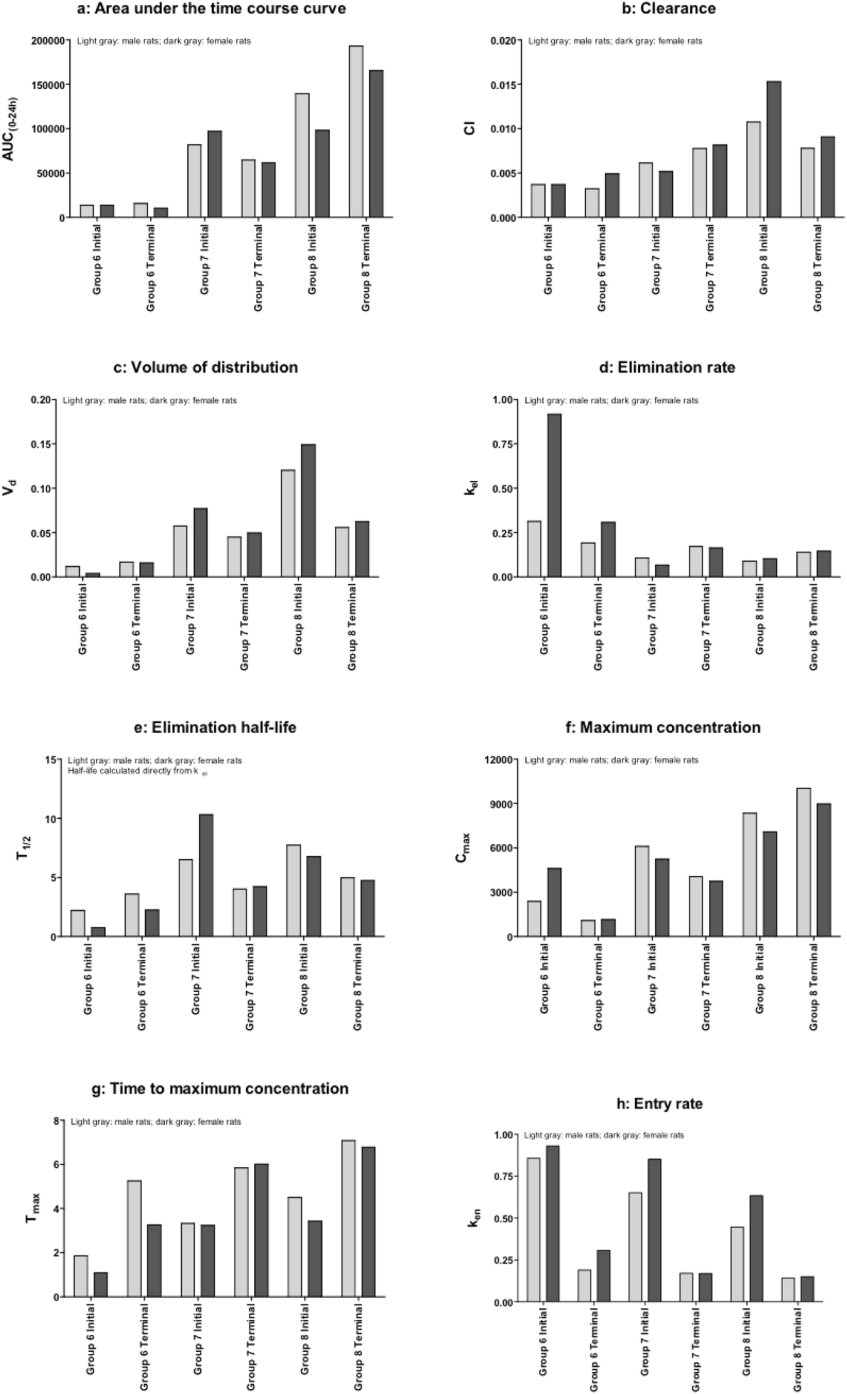
**(a**–**h**) - Bioanalytical parameters from satellite animals.

Time course data were analyzed using a one-compartment pharmacokinetic model. As samples were collected using cohorts, values for each cohort of three animals were averaged at each time point and treated as one series, Figure S1. Area under the curve values for the 24-hour period following dosing (AUC_(0–24)_) showed an increase with dose (Fig. 2a). However, this increase was not proportional to dose; the 10-fold dose difference between Groups 6 and 7 produced approximately a six-fold increase in AUC_(0–24)_, and the 3-fold difference between Groups 7 and 8 produced approximately a two-fold increase in AUC_(0–24)_. No consistent effect of phase (initial vs. terminal) or sex (male vs. female) was noted, although Group 8 AUC_(0–24)_ results did increase from initial to terminal values. A failure to observe dose-proportional AUC_(0–24)_ values can reflect clearance changes. In this study, however, differences in observed clearance rates (Fig. 2b) are modest, with slight increases for Group 8 but little difference between Groups 6 and 7. No consistent effect of phase or sex was noted. Clearance rates were determined by volume of distribution and elimination. Despite the modest differences in clearance rates, volume of distribution tended to increase with dose (Fig. 2c). In Groups 7 and 8, volume of distribution decreased during the course of the study. No clear effect of sex was noted. In contrast, elimination rate (k_el_) decreased with dose (Fig. 2d). In Group 5, elimination rate was higher in females than males; this difference was not observed in other groups. Elimination half-lives (T_1/2_), calculated directly from elimination rates, are presented in Fig. 2e.

Maximum plasma concentration (C_max_) tended to show the same pattern of results as AUC_(0–24)_, although the dose-proportionality of C_max_ was even more shallow (compare Fig. 2f with Fig. 2a). No clear effect of phase or sex was noted. Time to maximum plasma concentration (T_max_) tended to rise with dose and, in Groups 6 and 7, with phase (Fig. 2g). No clear effect of sex was noted. This was largely reflected in entry rates (k_en_); entry rates decreased with dose and, in all groups, with phase (Fig. 2g). No clear effect of sex was noted.

### Hematology and serum chemistry

Hematology findings consisted of decreases in white blood cells (7.80–9.45 × 10^3^/µL), neutrophils (0.97–1.13 × 10^3^/µL), lymphocytes (6.50–7.95 × 10^3^/µL), eosinophils (0.07–0.10 × 10^3^/µL) and basophils (0.01–0.03 × 10^3^/µL) were observed in male rats at all dose levels as compared to the vehicle control group’s white blood cells (12.33 × 10^3^/µL), neutrophils (1.39 × 10^3^/µL), lymphocytes (10.48 × 10^3^/µL), eosinophils (0.18 × 10^3^/µL) and basophils (0.05 × 10^3^/µL) (Table S4). These decreases reached statistical significance (p < 0.05) in white blood cells, lymphocytes and basophils for Group 3 and basophils for Group 4. A statistically significant increase (p < 0.05) in monocytes (0.37 × 10^3^/µL) was observed in Group 4 females when compared to the vehicle control group (0.16 × 10^3^/µL).

Serum chemistry changes were limited to increases in total cholesterol (87–97 mg/dL) and triglycerides (36–45 mg/dL) observed in Group 3 and 4 females, as compared to vehicle control group’s total cholesterol (58 mg/dL) and triglycerides (30 mg/dL) (Table S5). These increases reached statistical significance (p < 0.05) in total cholesterol for Group 3 and in total cholesterol and triglycerides for Group 4). Statistically significant decreases (p < 0.05) were observed in aspartate aminotransferase for Group 4 females (61 U/L), alkaline phosphatase for Group 3 and 4 females (115 and 113 U/L, respectively), and total bilirubin for Group 4 females (0.15 mg/dL), as compared to vehicle control group’s aspartate aminotransferase (93 U/L), alkaline phosphatase (158 U/L), and total bilirubin (0.19 mg/dL).

### Organ weights and tissue observations

There were no changes in absolute and relative organ weights attributable to Ramizol administration. No macroscopic observations were noted for any main animal during the study. There were no microscopic findings directly related to Ramizol administration. Two out of three females treated at 1500 mg/kg/day had evidence of minimal mixed inflammatory cells infiltrates in the stomach associated with minimal edema that were interpreted to be secondary to the presence and interaction of Ramizol with the stomach wall. There were no changes observed in other gastrointestinal tissues (i.e., cecum, duodenum, ileum, or colon).

## Discussion

The limited dose-proportionality of AUC_(0–24)_ and C_max_ values, combined with dose-dependent V_d_ and complex changes in entry rate, suggest that a distribution process is saturating; this could reflect saturation of a transport process, limited test article solubility in the GI tract or other issues.

Hematology findings consisted of decreases in white blood cells, neutrophils, lymphocytes, eosinophils and basophils in male rats at all dose levels that occasionally reached statistical significance in males given 500 mg/kg/day and/or 1500 mg/kg/day. These decreases were not dose-dependent and were within the historical control range, which suggest the changes are not treatment related. A statistically significant higher number of monocytes was observed in female rats administered the high dose of 1500 mg/kg/day. However, no corresponding changes were observed in the microscopic examination of the Group 4 bone marrow in either sex. Serum chemistry changes were limited to increases in total cholesterol and triglycerides observed in female rats given 500 mg/kg/day and 1500 mg/kg/day. These increases were in general of low magnitude, were within the historical control range, and did not correlate with any Ramizol related finding in the tissues examined. In order to establish the toxicological significance and adversity of these findings, the evaluation of a larger number of animals and a complete evaluation of systemic toxicity is warranted; however in this regimen of administration and without any correlating clinical signs, and/or macroscopic or macroscopic observations, these small magnitude changes in both hematology and serum chemistry, which are within the laboratory historical control values, did not apparently impact drug tolerability and are not considered adverse under the conditions of the study.

Under the conditions of this study and based on the toxicological endpoints evaluated, Sprague Dawley rats are expected to tolerate oral dose concentration of at least 1500 mg/kg/day of Ramizol for males and females in a study of 14 days or longer duration. Due to the high tolerability observed in this study at 1500 mg/kg/day, GLP studies will therefore use the limit dose (2000 mg/kg/day) or the maximum feasible dose as the high dose. In conclusion, Ramizol is an extremely well-tolerated antibiotic in rats, with good microbiology and antioxidant properties. Furthermore, Ramizol has high chemical stability and a scalable and low cost of manufacturing. These advantages might position it favourably in a CDI market with few treatment options.

## Electronic supplementary material

Supplementary Information
