# Supplementary material for: A 14-day repeat dose oral gavage range-finding study of a first-in-class CDI investigational antibiotic, in rats
Source: Sci Rep. 2019 Jan 17;9:158. doi: 10.1038/s41598-018-36690-9 (PMC6336794; doi:10.1038/s41598-018-36690-9)
Supplement: Supplementary file 1 — Supplementary Information [file 41598_2018_36690_MOESM1_ESM.docx]

**A 14-day repeat dose oral gavage range-finding study of a first-in-class CDI investigational antibiotic, in rats**

Katherine Sibley^1^, Jayson Chen^1^, Lee Koetzner^1^, Odete Mendes^1^, Amy Kimzey^2^, Janice Lansita^2^, and Ramiz A Boulos^,3,4^*

^1^Product Safety Labs, Dayton, NJ, United States of America

^2^ToxStrategies, Inc. Katy, TX, United States of America

^3^School of Chemical and Physical Sciences, Flinders University, Bedford Park, SA, Australia

^4^Antibiotic Development, Boulos & Cooper Pharmaceuticals Pty Ltd, Balcatta, WA, Australia

CEO@bouloscooper.com

1. **Table S1** - Chemical stability results of neat test substance samples

| **Sampling Day** | **Measured  AI (%)** | **% Change** | **Overall**  **Stability (%)** |
| --- | --- | --- | --- |
| Study Day 0 (Initial) | 104.6 | -0.4 | 99.6 |
| Study Day 6 | 104.1 |  |  |

1. **Table S2** - Chemical stability results for the dose preparation. Note: due to shortage in week 1 dose preparations, week 2 preparations were sampled and analyzed to evaluate the stability of the test substance preparations under refrigeration. The % of Target was calculated as [Calculated Test Substance in dose solution (mg/mL)/Target Dose Concentration (mg/mL)] x 100

| **Study Day** | **Group** | **Dose Level**  **(mg/mL)** | **Average Conc. (mg/mL)** | **% of Target** | **% Change** | **Overall Stability** |
| --- | --- | --- | --- | --- | --- | --- |
| 7 | 1 | 0 | ND | NA | NA | NA |
|  | 2 | 5 | 4.3 | 85.7 | NA | NA |
|  | 3 | 50 | 50.7 | 101.3 | NA | NA |
|  | 4 | 150 | 156.5 | 104.3 | NA | NA |
| 14 | 1 | 0 | ND | NA | NA | NA |
|  | 2 | 5 | 4.6 | 91.4 | 6.7 | 106.7 |
|  | 3 | 50 | 49.7 | 99.3 | -2.0 | 98.0 |
|  | 4 | 150 | 152.3 | 101.6 | -2.7 | 97.3 |

**ND = Not Detected; NA = Not Applicable**

1. **Table S3** - Chemical analysis for homogeneity and concentration verification dose preparation on day 1. The % Target was calculated as [Average measured concentration (mg/mL)/Target Concentration (mg/mL)] x 100.

| **Group** | **Target Conc.**  **(mg/mL)** | **Sampling Location** | **Average Measured Conc. (mg/mL)** | **% of Target** | **Average**  **% of Target** | **%RSD** |
| --- | --- | --- | --- | --- | --- | --- |
| 1 | 0 | Middle | ND | NA | NA | NA |
| 2 | 5 | Top | 5.2 | 103.2 | 105.5 | 2.2 |
|  |  | Middle | 5.3 | 105.4 |  |  |
|  |  | Bottom | 5.4 | 107.8 |  |  |
| 3 | 50 | Top | 56.6 | 113.1 | 111.1 | 1.7 |
|  |  | Middle | 55.4 | 110.8 |  |  |
|  |  | Bottom | 54.7 | 109.4 |  |  |
| 4 | 150 | Top | 151.2 | 100.8 | 101.5 | 0.7 |
|  |  | Middle | 152.3 | 101.5 |  |  |
|  |  | Bottom | 153.2 | 102.1 |  |  |

**ND = Not Detected; NA = Not Applicable**

**Table S4** – Summary of hematology values at Day 15 of the main test animals (n=3). An asterisk (*) denotes statistical significance using the Dunnett 2 Sided Test p<0.05.

|  |  | Group 1  0 mg/kg/day | | Group 2  50 mg/kg/day | | Group 3  500 mg/kg/day | | Group 4  1500 mg/kg/day | | Historical control data range^1^ | |
| --- | --- | --- | --- | --- | --- | --- | --- | --- | --- | --- | --- |
|  |  | Males | Females | Males | Females | Males | Females | Males | Females | Males | Females |
| Red Blood Cell Count (RBC) (x 10^6^/µL) | Mean | 7.47 | 7.57 | 7.38 | 7.73 | 7.44 | 7.37 | 7.25 | 7.44 | 5.07-10.04 | 6.99-9.34 |
|  | SD | 0.38 | 0.18 | 0.15 | 0.58 | 0.20 | 0.48 |  | 0.06 |  |  |
|  | %Diff | - | - | -1.2 | 2.1 | -0.5 | -2.6 | 3 | -1.7 |  |  |
| Hemoglobin (HGB) (g/dL) | Mean | 14.9 | 15.3 | 15.2 | 15.5 | 14.6 | 14.9 | -3.0 | 14.9 | 10.5-17.4 | 13.4-17.1 |
|  | SD | 0.7 | 0.6 | 0.7 | 0.6 | 0.5 | 0.8 | 14.2 | 0.4 |  |  |
|  | %Diff | - | - | 1.6 | 1.1 | -2.0 | -2.6 | -4.9 | -2.8 |  |  |
| Hematocrit (HCT) (%) | Mean | 45 | 43.5 | 45.2 | 45.1 | 43.3 | 43.2 | 42.6 | 43.2 | 34.8-51.1 | 38.0-49.4 |
|  | SD | 2.5 | 1.7 | 2.1 | 2.1 | 1.4 | 2.7 | 1.9 | 1.2 |  |  |
|  | %Diff | - | - | 0.4 | 3.7 | -3.8 | -0.8 | -5.3 | -0.7 |  |  |
| Mean Corpuscular (cell) volume (MCV) (fL) | Mean | 60.2 | 57.5 | 61.3 | 58.6 | 58.2 | 58.6 | 58.8 | 58.2 | 47.5-68.6 | 49.9-59.5 |
|  | SD | 1.6 | 0.9 | 2.4 | 1.7 | 1.4 | 0.5 | 1.3 | 1.4 |  |  |
|  | %Diff | - | - | - | 1.8 | -3.3 | 1.9 | -2.3 | 1.2 |  |  |
| Mean Corpuscular (cell) hemoglobin (MCH) (pg) | Mean | 20 | 20.2 | 20.6 | 20.1 | 19.7 | 20.3 | 19.6 | 20.0 | 15.3-21.5 | 16.9-20.8 |
|  | SD | 0.3 | 0.4 | 0.8 | 0.7 | 0.3 | 0.3 | 0.5 | 0.5 |  |  |
|  | %Diff | - | - | 3 | -0.5 | -1.5 | 0.3 | -2.0 | -1.0 |  |  |
| Mean Corpuscular (cell) hemoglobin concentration (MCHC) (g/dL) | Mean | 33.2 | 35.2 | 33.6 | 34.3 | 33.8 | 34.6 | 33.3 | 34.4 | 30.1-35.8 | 32.5-36.5 |
|  | SD | 0.4 | 0.6 | 0.3 | 0.3 | 0.5 | 0.5 | 0.6 | 0.2 |  |  |
|  | %Diff | - | - | 1.0 | -2.4 | 1.6 | -1.6 | 0.2 | -2.1 |  |  |
| Red Cell Distribution Width (RDW) (%) | Mean | 12.5 | 11.5 | 12.3 | 11.1 | 12.4 | 11.2 | 13.2 | 11.1 | 11.1-35.8 | 10.1-13.3 |
|  | SD | 0.2 | 0.3 | 0.5 | 0.5 | 0.5 | 0.4 | 0.4 | 0.1 |  |  |
|  | %Diff | - | - | -1.3 | -3.5 | -0.8 | -2.6 | 5.9 | -2.9 |  |  |
| Platelet Count (PLT) (x 10^3^/µL) | Mean | 1150 | 1257 | 1046 | 1231 | 1025 | 1103 | 1167 | 1084 | 393.0-1799.0 | 502.0-1594.0 |
|  | SD | 147 | 251 | 114 | 154 | 219 | 102 | 87 | 82 |  |  |
|  | %Diff | - | - | -9.0 | -2.1 | -10.9 | -12.3 | 1.5 | -13.7 |  |  |
| White Blood Cell Count (WBC) (x 10^3^/µL) | Mean | 12.33 | 9.23 | 9.45 | 8.52 | 7.80* | 9.40 | 9.08 | 12.26 | 6.19-23.97 | 2.41-17.26 |
|  | SD | 2.08 | 2.27 | 2.06 | 1.99 | 1.07 | 1.79 | 1.07 | 1.76 |  |  |
|  | %Diff | - | - | -23.4 | -7.7 | -36.7 | 1.9 | -26.4 | 32.9 |  |  |
| Absolute Neutrophil (ANEU) (x 10^3^/µL) | Mean | 1.39 | 1.01 | 1.13 | 0.89 | 1.03 | 1.21 | 0.97 | 1.28 | 0.53-9.39 | 0.31-4.08 |
|  | SD | 0.35 | 0.34 | 0.38 | 0.21 | 0.17 | 0.45 | 0.40 | 0.84 |  |  |
|  | %Diff | - | - | -19.1 | -12.4 | -25.8 | 19.5 | -30.0 | 26.2 |  |  |
| Absolute Lymphocyte (ALYM) (x 10^3^/µL) | Mean | 10.48 | 7.88 | 7.95 | 7.25 | 6.50* | 7.75 | 7.81 | 10.36 | 3.33-20.32 | 1.80-15.36 |
|  | SD | 1.66 | 2.53 | 1.76 | 1.57 | 0.89 | 1.36 | 0.65 | 1.75 |  |  |
|  | %Diff | - | - | -24.1 | -8.1 | -37.9 | -1.7 | -25.5 | 31.4 |  |  |
| Absolute Monocyte (AMON) (x 10^3^/µL) | Mean | 0.19 | 0.16 | 0.20 | 0.18 | 0.12 | 0.22 | 0.18 | 0.37* | 0.09-1.25 | 0.03-0.59 |
|  | SD | 0.06 | 0.06 | 0.05 | 0.12 | 0.03 | 0.03 | 0.05 | 0.05 |  |  |
|  | %Diff | - | - | 6.0 | 12.5 | -38.3 | 35.0 | -7.9 | 125.3 |  |  |
| Absolute Eosinophil (AEOS) (x 10^3^/µL) | Mean | 0.18 | 0.10 | 0.10 | 0.13 | 0.10 | 0.14 | 0.07 | 0.14 | 0.00-0.88 | 0.04-0.84 |
|  | SD | 0.11 | 0.04 | 0.01 | 0.07 | 0.03 | 0.07 | 0.03 | 0.04 |  |  |
|  | %Diff | - | - | -43.8 | 29.9 | -45.4 | 44.6 | -62.7 | 41.6 |  |  |
| Absolute Basophil (ABAS) (x 10^3^/µL) | Mean | 0.05 | 0.02 | 0.03 | 0.02 | 0.01* | 0.02 | 0.02* | 0.05 | 0.00-0.27 | 0.00-0.15 |
|  | SD | 0.01 | 0.02 | 0.02 | 0.02 | 0.01 | 0.01 | 0.01 | 0.02 |  |  |
|  | %Diff | - | - | -38.8 | 3.7 | -72.0 | -4.6 | -55.5 | 97.4 |  |  |
| Absolute Large Unstained Cell (ALUC) (x 10^3^/µL) | Mean | 0.05 | 0.05 | 0.04 | 0.05 | 0.03 | 0.06 | 0.04 | 0.08 | 0.00-0.47 | 0.00-0.26 |
|  | SD | 0.02 | 0.01 | 0.02 | 0.03 | 0.02 | 0.01 | 0.01 | 0.01 |  |  |
|  | %Diff | - | - | -10.3 | 12.8 | -31.4 | 35.9 | -17.3 | 70.9 |  |  |
| Absolute Reticulocyte Count (ARET) (x 10^3^/µL) | Mean | 302.1 | 198.4 | 281.6 | 212.0 | 273.2 | 218.8 | 324.7 | 183.2 | 121.4-1913.0 | 27.7-279.8 |
|  | SD | 20.7 | 25.0 | 26.7 | 26.9 | 18.8 | 2.6 | 60.9 | 12.3 |  |  |
|  | %Diff | - | - | -6.8 | 6.9 | -9.6 | 10.3 | 7.5 | -7.7 |  |  |

**Table S5** – Summary of clinical chemistry values at Day 15 of the main test animals (n=3). An asterisk (*) denotes statistical significance using the Dunnett 2-Sided Test p<0.5, ^ denotes groups with two values and # denotes groups with one value.

|  |  | Group 1  0 mg/kg/day | | Group 2  50 mg/kg/day | | Group 3  500 mg/kg/day | | Group 4  1500 mg/kg/day | | Historical control data range^1^ | |
| --- | --- | --- | --- | --- | --- | --- | --- | --- | --- | --- | --- |
|  |  | Males | Females | Males | Females | Males | Females | Males | Females | Males | Females |
| Asparate Aminotransferase (AST) (U/L) | Mean | 87^ | 93 | 109^ | 102 | 73^ | 79 | 92# | 61* | 53-740 | 46-460 |
|  | SD | 8 | 6 | 30 | 18 | 5 | 10 |  | 12 |  |  |
|  | %Diff | - | - | 25.4 | 9.6 | -16.2 | -15.0 | 6.4 | -34.3 |  |  |
| Alanine Aminotransferase (ALT) (U/L) | Mean | 33 | 31 | 37 | 36 | 32 | 30 | 32 | 25 | 18-473 | 13-283 |
|  | SD | 4 | 3 | 4 | 6 | 1 | 4 | 5 | 2 |  |  |
|  | %Diff | - | - | 13.3 | 16.3 | -3.1 | -1.1 | -1.0 | -17.4 |  |  |
| Sorbitol Dehydrogenase (SDH) (U/L) | Mean | 2.4^ | 6.5 | 3.5^ | 7.2 | 6.3^ | 7.3 | -5.1# | 6.9 | 0.0-141.0 | 0.2-42.7 |
|  | SD | 2.5 | 1.7 | 2.1 | 4.2 | 0.4 | 3.1 |  | 0.3 |  |  |
|  | %Diff | - | - | 45.8 | 11.3 | 160.4 | 13.4 | 112.5 | 7.2 |  |  |
| Alkaline Phosphates (ALKP) (U/L) | Mean | 187 | 158 | 169 | 131 | 184 | 115* | 205 | 113* | 46-742 | 19-297 |
|  | SD | 35 | 21 | 50 | 23 | 78 | 6 | 62 | 18 |  |  |
|  | %Diff | - | - | -9.4 | -17.3 | -1.6 | -27.6 | 9.4 | -28.4 |  |  |
| Total Bilirubin (BILI) (mg/d) | Mean | 0.16 | 0.19 | 0.16 | 0.17 | 0.19 | 0.16 | 0.18 | 0.15* | 0.08-0.29 | 0.8-0.28 |
|  | SD | 0.02 | 0.01 | 0.02 | 0.02 | 0.01 | 0.01 | 0.01 | 0.03 |  |  |
|  | %Diff | - | - | 0.0 | -8.8 | 14.3 | -15.8 | 10.2 | -21.1 |  |  |
| Urea Nitrogen (BUN) (mg/dL) | Mean | 12 | 12 | 11 | 13 | 11 | 11 | 9 | 11 | 6-46 | 7-21 |
|  | SD | 2 | 2 | 1 | 2 | 2 | 1 | 2 | 1 |  |  |
|  | %Diff | - | - | -11.1 | 5.4 | -8.3 | -10.8 | -25.0 | -8.1 |  |  |
| Creatinine (CREA) (mg/dL) | Mean | 0.29 | 0.34 | 0.26 | 0.32 | 0.29 | 0.31 | 0.26 | 0.30 | 0.13-159 | 0.17-0.53 |
|  | SD | 0.03 | 0.04 | 0.02 | 0.04 | 0.05 | 0.03 | 0.02 | 0.02 |  |  |
|  | %Diff | - | - | -10.2 | -4.9 | -2.3 | -7.8 | -10.2 | -12.7 |  |  |
| Cholesterol (CHOL) (mg/dL) | Mean | 68 | 58 | 65 | 71 | 80 | 87* | 80 | 97* | 34-159 | 36-225 |
|  | SD | 15 | 6 | 20 | 4 | 14 | 5 | 13 | 28 |  |  |
|  | %Diff | - | - | -4.4 | 21.1 | 17.7 | 49.7 | 18.7 | 66.9 |  |  |
| Triglycerides (TRIG) (mg/dL) | Mean | 56 | 30 | 46 | 28 | 36 | 36 | 54 | 45* | 18-291 | 16-282 |
|  | SD | 6 | 4 | 19 | 3 | 2 | 5 | 27 | 9 |  |  |
|  | %Diff | - | - | -17.4 | -7.8 | -35.3 | 20.0 | -2.4 | 51.1 |  |  |
| Glucose (GLUC) (mg/dL) | Mean | 115 | 105 | 115 | 119 | 125 | 107 | 110 | 111 | 68-264 | 74-174 |
|  | SD | 5 | 9 | 15 | 13 | 21 | 13 | 3 | 14 |  |  |
|  | %Diff | - | - | 0.0 | 13.7 | 8.7 | 1.9 | -4.3 | 5.7 |  |  |
| Total Protein (TP) (g/dL) | Mean | 6.0 | 6.1 | 5.8 | 6.2 | 5.8 | 6.2 | 5.7 | 6.2 | 4.9-7.4 | 5.1-8.9 |
|  | SD | 0.3 | 0.2 | 0.2 | 0.2 | 0.2 | 0.3 | 0.1 | 0.3 |  |  |
|  | %Diff | - | - | -3.9 | 2.7 | -3.9 | 1.6 | -5.5 | 1.6 |  |  |
| Albumin (ALB) (g/dL) | Mean | 3.4 | 3.4 | 3.3 | 3.5 | 3.2 | 3.5 | 3.2 | 3.5 | 2.6-3.9 | 2.9-5.2 |
|  | SD | 0.2 | 0.1 | 0.1 | 0.1 | 0.2 | 0.2 | 0.1 | 0.2 |  |  |
|  | %Diff | - | - | -4.9 | 1.0 | -5.5 | 2.9 | -5.8 | 1.9 |  |  |
| Globulin (GLOB) (g/dL) | Mean | 2.6 | 2.6 | 2.5 | 2.8 | 2.6 | 2.6 | 2.5 | 2.7 | 2.0-4.2 | 1.9-4.2 |
|  | SD | 0.2 | 0.3 | 0.2 | 0.2 | 0.1 | 0.2 | 0.1 | 0.1 |  |  |
|  | %Diff | - | - | -2.6 | 5.1 | -1.3 | 0.0 | -5.1 | 1.3 |  |  |
| Calcium (CALC) (mg/dL) | Mean | 9.9 | 9.6 | 10.0 | 9.7 | 9.7 | 9.8 | 9.7 | 10.3 | 8.1-11.9 | 8.7-12.1 |
|  | SD | 0.6 | 0.2 | 0.3 | 0.4 | 0.1 | 0.4 | 0.3 | 0.6 |  |  |
|  | %Diff | - | - | 0.3 | 1.0 | -2.3 | 1.7 | -2.3 | 7.3 |  |  |
| Inorganic Phosphorous (IPHS) (mg/dL) | Mean | 7.9* | 7.1 | 7.9# | 6.7 | 7.9# | 6.8 | 7.9# | 7.3 | 4.9-11.0 | 2.6-11.0 |
|  | SD | 1.7 | 0.8 | 0.1 | 0.2 | 0.4 | 0.3 |  | 0.3 |  |  |
|  | %Diff | - | - | -0.6 | -5.2 | -0.6 | -3.3 | 0.0 | 3.3 |  |  |
| Sodium (NA) (mmol/L) | Mean | 141.2 | 140.4 | 142 | 139.4 | 141.8 | 140.0 | 141.3 | 138.9 | 127.6-166.1 | 126.3-162.0 |
|  | SD | 1.9- | 1.9 | 1.2 | 0.8 | 1.4 | 0.8 | 1.1 | 0.9 |  |  |
|  | %Diff | - | - | 0.6 | -0.7 | 0.4 | -0.3 | 0.1 | -1.1 |  |  |
| Potassium (K) (mmol/L) | Mean | 5.31 | 4.62 | 5.14 | 4.84 | 5.14 | 4.74 | 5.15 | 4.74 | 3.97-8.46 | 3.52-7.69 |
|  | SD | 0.04 | 0.29 | 0.33 | 0.13 | 0.32 | 0.12 | 0.27 | 0.20 |  |  |
|  | %Diff | - | - | -3.3 | 4.8 | -3.2 | 2.7 | -3.1 | 2.6 |  |  |
| Chloride (CL) (mmol/L) | Mean | 103.9 | 102.9 | 104.1 | 103.7 | 103.1 | 103.4 | 103.2 | 101.8 | 91.9-120.9 | 92.9-117.9 |
|  | SD | 0.6 | 1.9 | 0.5 | 1.0 | 1.2 | 0.9 | 1.2 | 1.3 |  |  |
|  | %Diff | - | - | 0.3 | 0.8 | -0.8 | 0.4 | -0.6 | -1.1 |  |  |

**REFERENCES**

1. PSL historical control data from CD(SD) rats at approximately 11-22 weeks of age; April, 2012 – November, 2017.
